# Supplementary material for: The interactome of intact mitochondria by cross-linking mass spectrometry provides evidence for coexisting respiratory supercomplexes
Source: Mol Cell Proteomics. 2017 Dec 8;17(2):216–32. doi: 10.1074/mcp.RA117.000470 (PMC5795388; doi:10.1074/mcp.RA117.000470)
Supplement: Supplemental Data [file supp_RA117.000470_133922_0_supp_23978_fzffwf.zip › spectra_annotation/mito_DR_spectra_annotation/41-1-20-1-2-1.pdf]

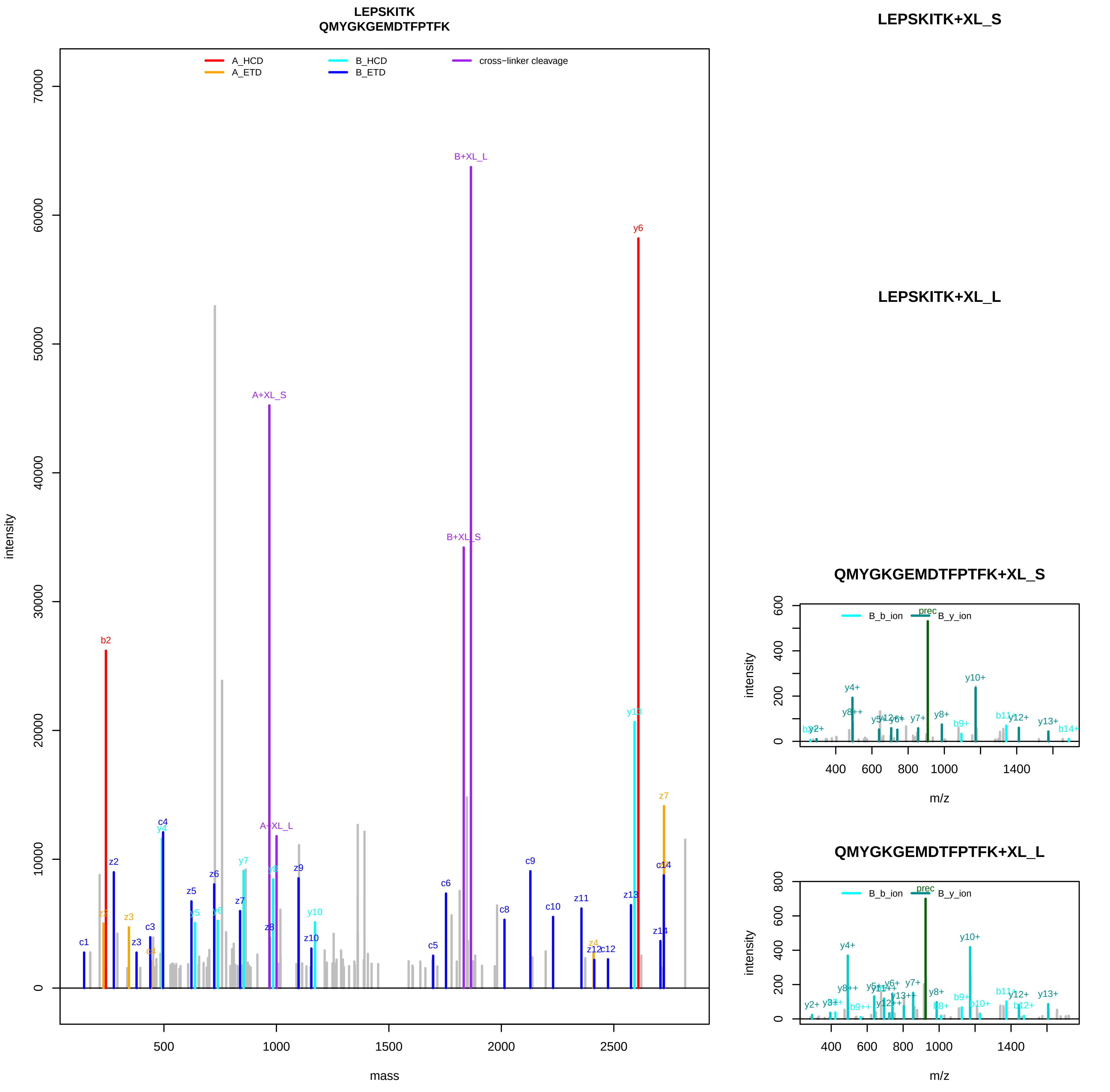
LEPSKITK
QMYGKGEMDTFPTFK
A_HCD
A_ETD
B_HCD
B_ETD
cross−linker cleavage
intensity
mass
B+XL_L
A+XL_S
B+XL_S
A+XL_L
y6
b2
y13
z7
c14
z14
z13
z11
c12
z12
z4
c10
c9
c8
c6
c5
z10
y10
z9
y8
z8
y7
z7
y6
z6
z5
y5
c4
y4
c3
c4
z3
z2
c1
z3
z2
LEPSKITK+XL_S
LEPSKITK+XL_L
QMYGKGEMDTFPTFK+XL_S
B_b_ion
B_y_ion
prec
y4+
y8++
y10+
y7+
y8+
b9+
y12+
y13+
b14+
b11+
m/z
QMYGKGEMDTFPTFK+XL_L
y10+
y7+
y6+
y8+
b9+
b10+
y12+
y13+
b11+
y2+
b9++
m/z
